# Dental Education

**Published:** 1884-06

**Authors:** 


### DENTAL EDUCATION.

We publish the article under this head after holding it over a month, because such specific charges have been challenged on all sides, and because it comes from a responsible source. Concerning the matter animadverted upon, the editor and publishers of this journal have no personal knowledge. If there be misstatements, our pages are open for their disproval. But as they are matters of common report, we think it in the interest of all parties that a chance should be given to those who are accused of irregular practices to meet the charges in an open manner, rather than that they should be circulated covertly, with no opportunity for defense. More than one college is charged with graduating students irregularly, and we hope that those who have any personal knowledge of cases will come to the front with the testimony, and give the accused an opportunity to justify themselves if they can.

Of some of those who are charged with receiving diplomas irregularly issued we *have* personal knowledge. Drs. Barlow and Meeker are known to be competent practitioners and reputable men. We do not understand that they, or the others whose names are mentioned, are charged with anything dishonorable. But their names could not be withheld without suppressing the article entirely, and that we do not think the best interests of the profession, or indeed of the Baltimore college, demand.

